# UQlust: combining profile hashing with linear-time ranking for efficient clustering and analysis of big macromolecular data

**DOI:** 10.1186/s12859-016-1381-2

**Published:** 2016-12-28

**Authors:** Rafal Adamczak, Jarek Meller

**Affiliations:** 1 Department of Informatics, Faculty of Physics, Astronomy and Informatics, Nicolaus Copernicus University, Grudziadzka 5, 87-100, Torun, Poland; 2 Departments of Environmental Health and Electrical Engineering & Computing Systems, University of Cincinnati, Cincinnati, USA; 3 Division of Biomedical Informatics, Cincinnati Children’s Hospital Medical Center, Cincinnati, USA

**Keywords:** Protein structure, RNA structure, Profile hashing, Hierarchical clustering, Model quality assessment, Macromolecular structure analysis

## Abstract

**Background:**

Advances in computing have enabled current protein and RNA structure prediction and molecular simulation methods to dramatically increase their sampling of conformational spaces. The quickly growing number of experimentally resolved structures, and databases such as the Protein Data Bank, also implies large scale structural similarity analyses to retrieve and classify macromolecular data. Consequently, the computational cost of structure comparison and clustering for large sets of macromolecular structures has become a bottleneck that necessitates further algorithmic improvements and development of efficient software solutions.

**Results:**

uQlust is a versatile and easy-to-use tool for ultrafast ranking and clustering of macromolecular structures. uQlust makes use of structural profiles of proteins and nucleic acids, while combining a linear-time algorithm for implicit comparison of all pairs of models with profile hashing to enable efficient clustering of large data sets with a low memory footprint. In addition to ranking and clustering of large sets of models of the same protein or RNA molecule, uQlust can also be used in conjunction with fragment-based profiles in order to cluster structures of arbitrary length. For example, hierarchical clustering of the entire PDB using profile hashing can be performed on a typical laptop, thus opening an avenue for structural explorations previously limited to dedicated resources. The uQlust package is freely available under the GNU General Public License at https://github.com/uQlust.

**Conclusion:**

uQlust represents a drastic reduction in the computational complexity and memory requirements with respect to existing clustering and model quality assessment methods for macromolecular structure analysis, while yielding results on par with traditional approaches for both proteins and RNAs.

**Electronic supplementary material:**

The online version of this article (doi:10.1186/s12859-016-1381-2) contains supplementary material, which is available to authorized users.

## Background

Clustering techniques are widely used in the analysis and interpretation of molecular simulations for biological macromolecules, such as proteins and nucleic acids. For example, Markov state and related approaches for conformational space partitioning [1] are being used to analyze trajectories generated by Molecular Dynamics, e.g., to identify important folding intermediates [2]. Clustering is also used, either explicitly or implicitly, in order to identify high quality models generated by protein or RNA structure prediction methods [3]. In particular, geometric consensus methods for Model Quality Assessment (MQA) rank models by comparing their pairs, e.g., by 3D superposition, to find frequently sampled and hence likely well predicted substructures [4].

A number of methods have been developed recently to enable fast ranking and clustering of very large sets of protein models that can be generated by using current hardware in conjunction with simulation and structure prediction methods. Assessing structural similarity for pairs of models constitutes the major computational bottleneck in clustering and consensus-based MQA methods. Consequently, many ranking and clustering methods attempt to simplify structure-to-structure comparison to avoid 3D superposition, e.g., by projecting the structure into a structural motif frequency profile using a fragment library [5], by using parallelization to speed-up the loop over pairs of models [6], or by relying on traditional K-means clustering to limit the computational complexity [7], as only the distances to K centroids need to be computed.

For example, Pleiades [7] uses a projection of the backbone into 21-dimensional vectors using Gauss integrals, resulting in a fast K-means-based algorithm that compares structures in terms of their 21-dimensional backbone profiles. The FragBag approach [5], on the other hand, projects 3D structures into a frequency profile to enable fast structure-to-structure comparison and similarity search with a representative library of structural fragments. PconsD [8], a successful MQA method, computes model ranking by assessing similarity between protein models in terms of their distance matrices while using GPUs to speed-up comparison of all pairs of models. Parallelization is also used in ClusCo [6] to enable large scale all-vs-all comparison and enhance both hierarchical and K-means clustering in conjunction with the traditional Root Mean Square Deviation (RMSD) superposition.

Importantly, since K-means approaches do not imply a loop over pairs of models, the overall computation can be broken into data ‘slices’ and therefore easily implemented on modern distributed computing platforms for ‘big data’, such as Hadoop or Spark implementations of the Map/Reduce framework. In contrast, traditional hierarchical clustering techniques require that all pairs of structures are compared, making it incompatible with these distributed platforms. In addition, the overall distance matrix needs to be stored in memory, greatly limiting the size of data that can be analyzed using traditional hierarchical clustering approaches. Here, we address both of these challenges by combining structural profiles with a linear time geometric consensus-based ranking algorithm and profile hashing.

As shown in [9], by projecting 3D coordinates into a suitable 1D structural profile that assigns each residue to a distinct state, e.g. exposed vs. buried, the 1D-jury method implicitly compares all pairs of models to identify those that share common substructures without the need to perform a loop over pairs of models (see Additional file 1: Figure S1). Thus, 1D-jury provides geometric consensus-based ranking of all models in a set with a linear time complexity algorithm. In addition, 1D-jury provides natural centroids of clusters consisting of models sharing common substructures. It should be also noted that the 1D-jury approach can be generalized to arbitrary structures (of any length) as long as they can be projected into fixed length structural profiles, such as frequency profiles used by FragBag or Gauss integral projections used by Pleiades.

**Fig. 1:**
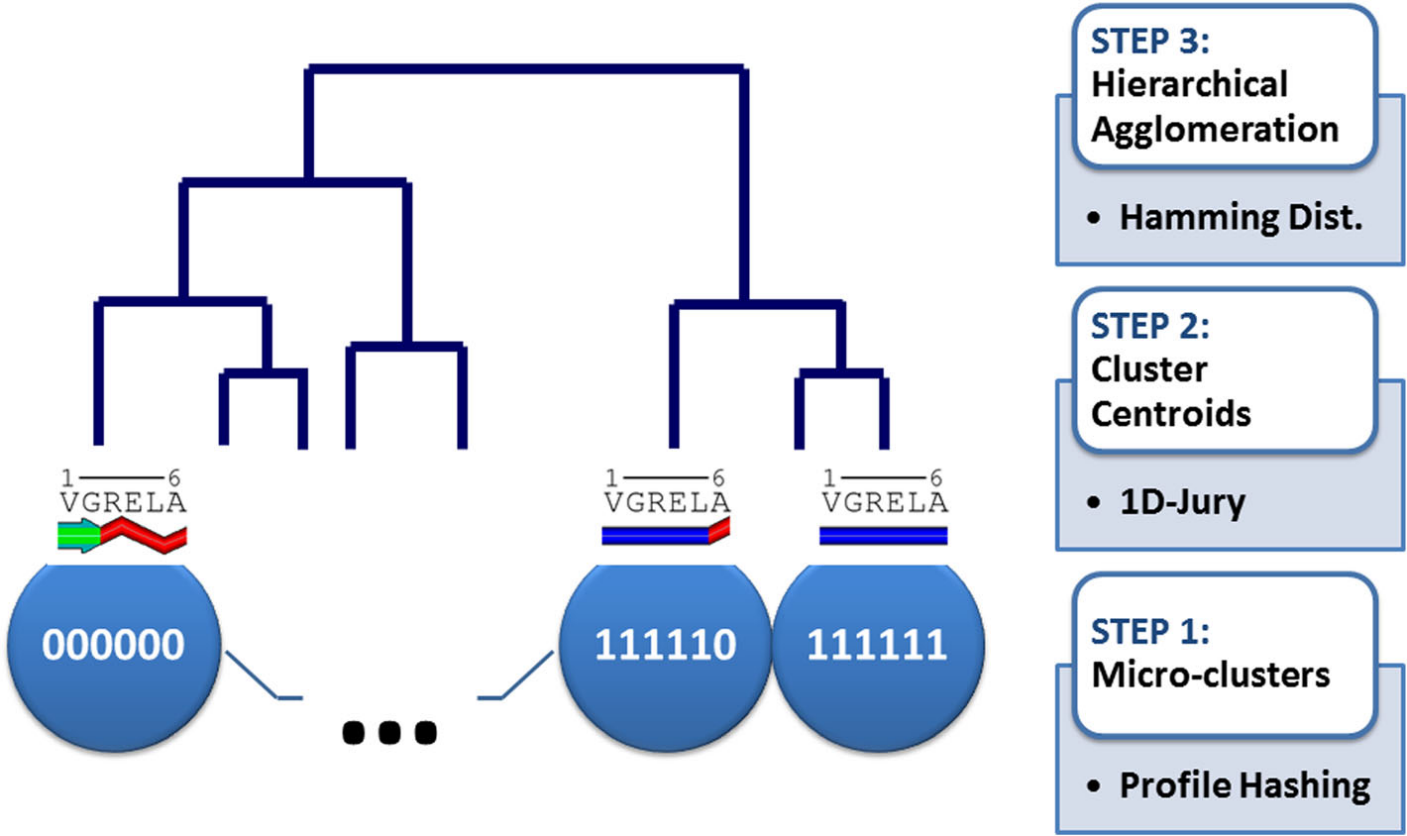
Schematic representation of approximate hierarchical clustering with profile hashing to generate ‘micro-clusters’ (lower level in the figure, with hashing keys in terms of consensus structure) that are subsequently hierarchically clustered, starting from the representative structures (1D-jury centroids shown above) in each micro-cluster, using an applicable distance measure, such as Hamming, cosine or RMSD (if 3D structures are available)

Structural profiles in conjunction with linear time consensus ranking can be further combined with profile hashing to enable efficient hierarchical clustering with a low memory footprint. The main idea is to use structural profiles in order to define hashing keys that map similar structures into the same values of a hash function, and thus enable collating profiles/structures with the same keys into initial micro-clusters. These micro-clusters are subsequently either tuned (with some level of profile coarse graining and further projections/filters) to obtain a certain number (K) of clusters and data coverage (the fraction of structures included in these K clusters), or aggregated hierarchically using the Hamming, cosine or other applicable distance measure (see Fig. 1). Building on these algorithmic engines, we present the uQlust package which combines 1D structural profiles, hashing and linear time ranking to enable ultrafast clustering of very large sets of atomistic or coarse-grained protein or RNA structures.

## Implementation

### Structural profiles

The following versatile residue-level projections of 3D structures (starting from a set of all-atom or reduced PDB or DCD files) are implemented in uQlust:

i) SS-SA or secondary structure (SS) – solvent accessibility (SA) profile, which assigns each amino acid residue to one of three secondary structures (N_SS_ = 3), and one of up to N_SA_ = 10 solvent accessibility states; the DSSP utility [10] is integrated with uQlust to assign SS and RSA states;
ii) CA(SS)-NC(SA) or approximate distance dependent secondary structure (SS) – solvent accessibility (SA) profile, which can be used for C_α_ only models, and assigns pseudo-secondary structure states based on distances between C_α_ atoms (CA) – for details see Supplementary Materials and uQlust Manual;
iii) CA-CM (contact map), which is also applicable to both atomistic and reduced models, and consists of the top triangle of the binary contact map, where d(C_α,i_,C_α,j_) < 8.5 Ang, |i-j| > 11.

Analogously to protein profiles, 1D RNA profiles for ranking and clustering (of equal length RNA models) are built either using a backbone phosphorus atom contact map (denoted as RNA-P-CM) where d(P_i_,P_j_) < 15.5 Ang, |i-j| > 11, or by considering a combination of secondary structure and base pairing states generated by using RNAview [11]. Namely, a simplified secondary structure assignment (stem vs. loop, N_SS_ = 2) is combined with a coarse-grained Leontis and Westhof (LW) classification of base-pairs into one of 15 different types based on nucleotide pairs (AT vs. GC), glycosidic bond orientation (cis vs. trans), interacting edges (Watson-Crick, Hoogsteen, Sugar Edge, and their frequently observed combinations plus ‘Other’ state), resulting in 30 distinct states (denoted as RNA-SS-LW).

Another type of profile available in uQlust uses a structural motif/fragment frequency profile to represent arbitrary structures (of any length). For proteins, uQlust uses the FragBag library [5] of 400 backbone fragments of length 11 residues, while its custom developed RNA-FragBag counterpart is used for RNAs (see the Results section).

A user-defined, protein or RNA, residue level or fragment-based profile can also be used in uQlust, in conjunction with an external application, such as DSSR [12]. A pre-defined workflow (denoted as RNA-SS-TA) combines simple secondary structure state assignment (N_SS_ = 2) with distinct torsional angle states (N_TA_ = 5), defined as combinations of DSSR epsilon-zeta BI and BII backbone states with chi syn- and anti- states (plus ‘other’ state). The resulting 10 distinct states can be further split based on base-pair type assignment, similar to that used for RNA-SS-LW.

Such defined profiles, as listed in Additional file 1: Table S1, can be used for either model assessment using 1D-Jury (denoted as uQlust:1D-ProfileName), or explicit clustering with profile hashing, using hash keys generated with a profile of choice to provide an initial ‘slicing’ of data.

### Profile hashing

Profile hashing is used in conjunction with 1D-Jury to achieve ultrafast clustering heuristics with a low memory footprint. Binary hash keys are generated with a 1D profile of choice by comparing each profile with a reference profile that obtains the maximum 1D-Jury score (which can be computed in linear time). The hash key for a profile is defined at each position as follows: 0 is added to the key if a given profile is in the same state as the reference profile at that position, 1 is added otherwise (note that the number of ones in a key is equal to the Hamming distance from the reference profile). Since the best 1D-jury score reference profile is expected to represent a natural geometric consensus for a substantial subset of models [9], one can also expect that many of such largely consistent models will likely obtain the same hash key, resulting in less granular partitioning into micro-clusters (subsets of profiles with the same value of the hashing function) compared with a random reference structure, or a direct use of multi-state profile as opposed to binary ‘geometric consensus’ keys.

### Clustering heuristics for big macromolecular data

The first heuristic discussed here is a profile hashing-based clustering that directly draws from the above considerations. It is referred to as uQlust:Hash (K,F), where K defines the number of target clusters, and F denotes the fraction of data that should be contained within those K clusters. Hash (K,F) starts by slicing data into micro-clusters with the same hashing function value. Subsequent agglomeration into K clusters (comprising F% of data) is obtained by simply changing the granularity of hash keys, which is achieved by removing a sufficient number of the most variable profile hash key positions characterized by high entropy across all data vectors.

Another heuristic in uQclust is a form of reference-based partitioning, which is referred to as uQlust:Rpart (K,F). As before, this new heuristic relies on the initial identification of 1D-jury ‘centroid’ for the entire data set, as a suitable reference conformation. For efficiency and granularity, Rpart also represents all profiles in terms of binary hash keys. However, the subsequent partitioning of data proceeds very differently. Rather than aggregating hashing-based micro-clusters, Rpart recursively identifies macro-clusters centered on a reference profile by adjusting the radius of clustering to achieve K clusters comprising F% of data.

Specifically, Hamming distances to the reference profile hash key are computed to identify a central inner sphere that contains data points closer than the radius of clustering (initially set to ¼ of the maximum distance from the 1D-jury reference) to the reference vector. Such defined sphere constitutes a candidate for a macro-cluster. The profile (structure) with the highest 1D-Jury score in the outer layer is then selected as the next reference structure. Next, the radius of clustering is reset to its distance from the original 1D-jury reference, and the process is repeated on the remainder of the data, considering only points at distances less than twice the current radius of clustering from the original 1D-jury reference. If after K iterations less than F% of data points are covered by such defined K candidate macro-clusters, then the size of the radius of clustering is increased (or decreased if more than F% of data points are covered), and the process is repeated. The process stops when no further improvement towards the targeted F is observed.

Finally, in the case of approximate hierarchical clustering, which is referred to as uQlust:Tree, the first step is analogous to that used for Rpart (K,F) or Hash (K,F), except that a large K is used to induce a large number of small clusters (micro-clusters, see Fig. 1) and F is set to 100% to include all data. While K can be modified by the user to set the tradeoff between speed and accuracy, its default value is set to *K* = 1,000 to provide sufficient granularity in both conformational space partitioning and model clustering for quality assessment (note that K and F are effectively fixed and can be dropped in references to uQlust:Tree). In the next step, a 1D-jury centroid is computed for each micro-cluster, and from this level traditional average distance agglomerative (bottom-up) hierarchical clustering with either Hamming or cosine distance (for arbitrary profiles), or RMSD (only for proteins or RNAs) can be applied. As a result, effectively linear complexity in the number of structures, N_struct_, is achieved when N_struct_ > > *K* (see running times in Table 2).

### Implementation details

uQlust is written in C# and should be easily portable between different operating systems (system independent pre-compiled executables that require .NET ver. 4.5 or higher, or Mono ver. 3.8 on 64-bit Windows or Linux operating systems, respectively, are provided with the distribution). Multithreading is implemented to speed-up profile pre-processing, ranking and clustering. Fast methods for RMSD [13] and MaxSub [14] structure similarity measures are implemented to speed-up structure to structure comparison when profiles are not used. For vector hashing, C# Dictionary Type with a hash function default method GetHashCode() is used. Work is in progress to enable the use of uQlust (in particular, for profile pre-processing) in conjunction with Hadoop Map/Reduce framework, using the Microsoft Azure plugin for C#.

## Results and discussion

### Linear time ranking of macromolecular models

As shown in [9], by projecting macromolecular 3D coordinates into a suitable 1D profile and profile pre-processing to compute the state frequency vector at each profile position, one can implicitly compare all pairs of models to compute their overall geometric consensus ranking with a linear time complexity algorithm. The resulting 1D-Jury approach enables ultrafast ranking of large sets of models, while yielding results on par with quadratic complexity methods, such as 3D-Jury [4] or PconsD [8]. This is illustrated in Additional file 1: Figure S1.

Here, uQlust is evaluated in terms of ranking and model assessment using CASP10 [15] and TASSER [16] benchmarks for proteins. Only those targets/models that were successfully processed by all methods are used for comparison (73 and 56 targets, and a total of 28,150 and 1,065,345 models, for CASP and TASSER respectively). Several well performing profiles, including a simple 1D-SS-SA and a contact map profile 1D-CA-CM, motivated by the success of PconsD (and to provide its linear complexity counterpart), are assessed.

As can be seen from Table 1, the running times indeed scale linearly with the number of structures for uQlust-1D-CA-CM, as opposed to quadratic scaling for PconsD. Furthermore, as can be seen from Table 2, the results of uQlust-1D-CA-CM and more compact uQlust-1D-SS-SA profile based ranking are on par with PconsD in terms of selection of top models. Interestingly, using centroids of explicitly identified clusters as top models leads to further improvements, especially for hashing and reference-based uQlust heuristics that outperform K-means approaches on CASP, while hierarchical uQlust:Tree clustering works best on TASSER.

**Table 1:** Running times for model ranking on TASSER target 256b_A

N_struct	2000	4000	8000	16,000
**Profile preprocessing**	13.8	51.6	132.0	231.0
**uQlust:1D-CA-CM**	0.6	1.2	3.0	6.6
PconsD	23.6	64.8	260.7	901.7

Time in CPU sec on a server with 8 Intel (R) Core (TM)2 Q6600@2.0GHz CPUs, 4 GB, and Linux version Ubuntu 12.04. PconsD was allowed to use all 8 CPUs and the TESLA C2075 graphical card with 448 GPUs, while times for uQlust are for 1 CPU only to demonstrate its linear scaling

**Table 2:** Evaluation of protein model quality assessment approaches

Method	CASP10	TASSER
PconsD	0.68 / 0.43	4.3 / 0.46
**uQlust:1D-CA-CM**	**0.66 / 0.38**	**4.2 / 0.46**
**uQlust:1D-SS-SA**	**0.67 / 0.40**	**4.3 / 0.41**
ClusCo (10)	0.68 / 0.37	3.2 / 0.49
Pleiades (10)	0.67 / 0.38	3.1 / 0.45
**uQlust: Hash (10,60)**	**0.76 / 0.52**	**3.5 / 0.44**
**uQlust: Rpart (10,60)**	**0.75 / 0.56**	**3.3 / 0.42**
**uQlust:Tree**	**0.71 / 0.46**	**2.9 / 0.47**

Average MaxSub similarity score between top ranking and best models (left), and fraction of good models (right) are reported for both CASP and TASSER targets. The fraction of good models is defined as the fraction of targets with the top ranking model less than 0.2 MaxSub score from the best model for CASP, and less than 2 Ang RMSD for TASSER. Centroids of the 5 largest (out of *K* = 10) clusters are considered for clustering methods, and *F* = 60% of data is used for uQlust

### Ultrafast clustering with profile hashing

Traditional and profile hashing-based hierarchical clustering techniques are compared in terms of time and memory usage in Table 3. We used coarse-grained models generated using the CABS-flex server [17] for three distinct conformers of Troponin C, increasing the number of models for each conformer to obtain a series of data sets of growing size, each consisting of 3 distinct clusters of equal number of structures. Note that, unlike for other hierarchical clustering methods tested, the running time and memory usage grow essentially linearly with the size of the problem for uQlust:Tree (here with the CA-CM profile, Rpart (1000,100) to define initial micro-clusters). Running times and memory allocation can be further reduced by replacing the CA-CM profile used here with a much more compact SS-SA profile.

**Table 3:** Time and memory usage for hierarchical clustering methods

N_struct	9000	18000	36,000	72,000	144,000
**Time (uQlust:Preprocess)**	**104**	**241**	**581**	**1604**	**3743**
**Time (uQlust:Tree)**	**70**	**92**	**168**	**310**	**488**
Time (ClusCo)	360	3080	24818	209072	---
Time (MaxClust)	7140	50540	---	---	---
**Memory (uQlust:Tree)**	**0.3**	**0.6**	**0.8**	**2.6**	**4.3**
Memory (ClusCo)	0.4	1.6	6.5	25	---
Memory (MaxClust)	1.9	5.7	19.0	---	---

CPU times (sec) and memory usage (GB) for approximate uQlust:Tree vs. full hierarchical clustering, obtained by using ClusCo [6] or MaxClust [22]. All calculations were performed on a server with 16 Intel (R) Xeon (R) E5-2680-0@2.70GHz CPUs, 132GB, and Linux version 2.6.32-504.1.3.el6.centos.plus.x86_64

It should be emphasized that there is a very high degree of concordance between full hierarchical and CA-CM (or SS-SA) profile-based uQlust:Tree, as indicated by the Rand index of 0.99 (at the level of 5 clusters) and illustrated in Additional file 1: Figure S3. Very similar results can also be obtained (data not shown) using the compact pseudo-secondary structure/contact number profile, CA(SS)-NC(SA), illustrating the versatility of uQlust. It should be also noted that RMSD can also be used in uQust:Tree to aggregate micro-clusters, providing in this case virtually identical results to those of full hierarchical clustering, without affecting significantly running times since only representative structures need to be superimposed.

In addition to applications to large scale structure prediction and molecular simulations of a target protein or RNA (of some fixed length), uQlust can also be used in conjunction with the FragBag structural motif frequency profile [5] for analysis and clustering of arbitrary structures (of different length), including an interactive exploration of the entire PDB. This is illustrated by performing hierarchical clustering of about 98,000 protein chains from the PDB that were assigned no more than one CATH fold level annotation to avoid ambiguous class assignment.

In order to perform the task on a Linux Intel i7-30610QM 2.3 GHz 4 core laptop, uQlust:Tree requires less than 2.5 GB of RAM and about 3.3 and 1.3 CPU hours for profile generation and hierarchical clustering, respectively, using the FragBag profile, Rpart (10000,90) micro-clusters and cosine similarity measure. For comparison, a simple hashing-based clustering with uQlust: Hash (10000,90) takes only about 30 CPU seconds, resulting in small clusters largely consistent at the superfamily level (but it obviously does not provide the overall hierarchical structure). It should be also noted that cosine similarity is more appropriate for frequency profiles that are characterized by large differences in counts and the overall profile vector norm, as opposed to Hamming distance that works well for residue level or contact map profiles (see Additional file 1: Figure S3 for instance).

**Fig. 2:**
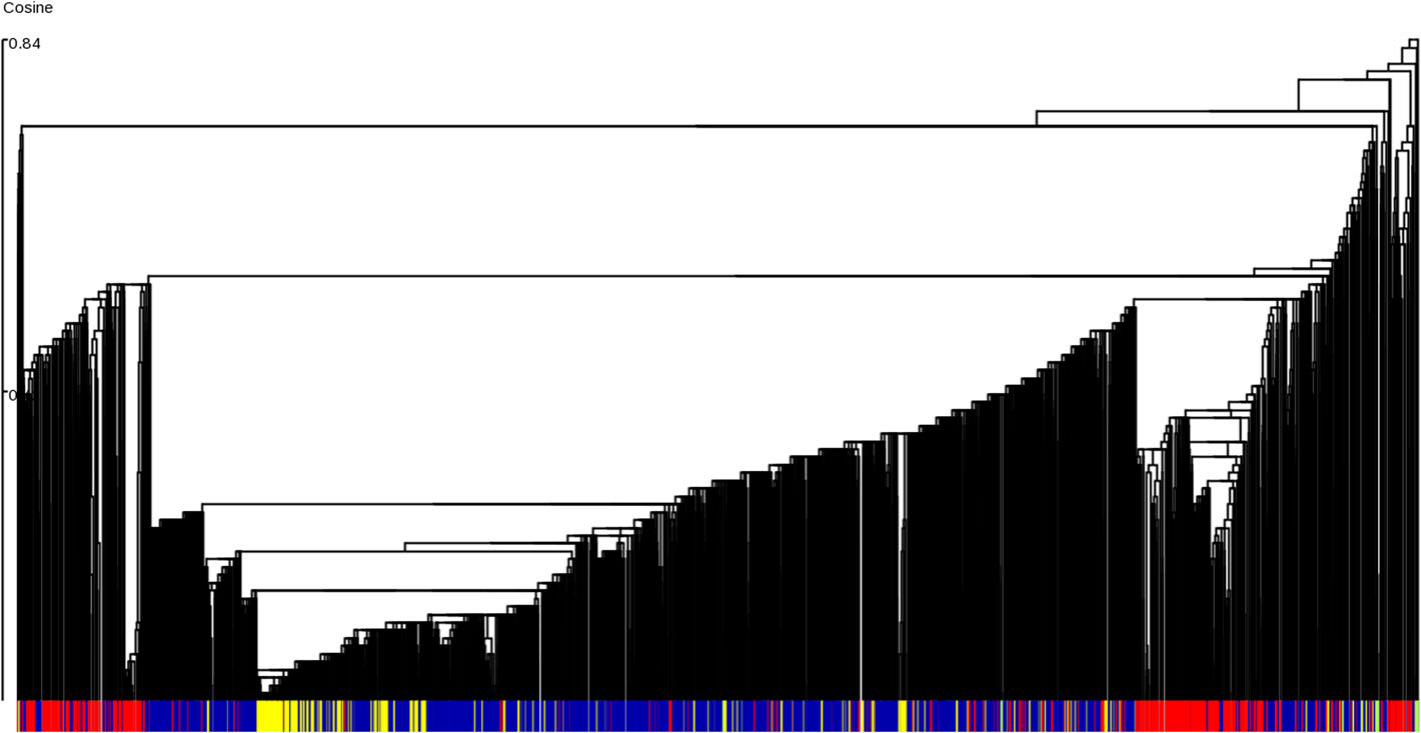
Hierarchical clustering of 98,000 protein chains from the Protein Data Bank, using the fragment-based FragBag profile and the uQlust:Tree algorithm. The initial micro-clusters of structures deemed as closely related (i.e. those with identical hash keys, including large “micro-clusters” of nearly identical structures such as those of globins or lysozymes) constitute the leaves in the tree. CATH assignment at the class level for majority alpha, alpha/beta (or alpha + beta) and beta clusters are shown as red, blue and yellow bars, respectively. It should be noted that the uQlust graphical user interface enables interactive exploration of such generated dendograms and other representations of large data sets

Note that CATH contains over thousand folds and about 2,700 superfamilies, requiring a large number (10,000) of micro-clusters for these highly granular data [18]. The results are illustrated in Fig. 2: the three main classes of proteins (alpha, alpha-beta and beta) cluster largely together, while within the micro-clusters the majority voting results in over 91%, 88% and 87% classification accuracy (that can be further improved to 96, 94 and 90% by increasing by 2-fold the number of micro-clusters at the expense of computation time) at the level of CATH class, architecture, and fold assignment, respectively.

Thus, despite large differences in length and significant variation in the secondary structure content within CATH fold or superfamily members, a simple FragBag profile can capture important global characteristics of the hierarchy of protein folds. On the other hand, the FragBag library had been primarily designed to enable fast retrieval of similar structures from PDB, rather than the overall structural classification. Importantly, the resolution and accuracy achieved here can be further improved by combining FragBag with other types of profiles, such as the Gauss integral representation of the backbone trace used by Pleiades [7]. Such extensions can be implemented by using a user defined profile in uQlust, and will be a subject of a future investigation.

### Clustering and ranking of RNA structures

We briefly illustrate the use of uQlust to cluster and rank RNA structures, using the FARNA benchmark [19], and a set of 23S, 16S and 5S ribosomal RNAs, collected from the SCOR database [20] and augmented by additional structures obtained from PDB. The results for six FARNA targets, with a subset of 500 models each, obtained using clustering to identify centroids of 5 largest clusters as top scoring models, are summarized in Table 4. The performance of uQlust: Rpart (10,60) with either RNA-SS-LW or RNA-SS-TA (only results for the former are shown) profile and Hamming distance are on par (or better) compared to enhanced K-means approach (uQust:10-means) with 3D structures and RMSD.

**Table 4:** Clustering-based RNA model quality assessment for FARNA

Target	Best RMSD	10-means (3D)	Rpart (10,60)
2a43	4.5	5.3	4.6
1a4d	3.8	11.9	6.0
1esy	2.9	3.4	3.3
1kka	3.6	4.5	4.5
1l2x	3.9	4.8	4.0
1q9a	4.1	4.4	4.7

RMSD (Ang) between the native structure and the closest of top 5 centroids, obtained using uQlust:K-means with RMSD distance (third column) or uQlust:Rpart with Hamming distance and RNA-SS-LW profile (last column), are compared with the best possible prediction, i.e., RMSD for the best model in a subset of 500 decoys for each target from [19]

Hierarchical clustering of ribosomal RNAs using a fragment-based profile is illustrated in Additional file 1: Figure S4. A library of 92 representative coarse-grained 5-mer backbone (phosphorus atom) RNA fragments, derived from the RNA05 set of RNA structures [21] is used to define an RNA fragment-based profile, denoted as RNA-FragBag. Such defined profile is then used in conjunction with uQlust:Tree and cosine distance, demonstrating high concordance with the three classes of ribosomal RNAs included.

Finally, we would like to emphasize that by enabling large-scale numerical experiments and benchmarking, uQlust can provide a platform for further refinement of profile-based approaches for macromolecular structure analysis and modeling, including the development of comprehensive RNA fragment libraries.

## Conclusions

By combining profile hashing in conjunctions with 1D residue level, fragment-based or arbitrary user defined profiles of proteins and RNAs, as well as the 1D-jury linear time complexity ranking algorithm with implicit comparison of all pairs of models [9], uQlust enables ultrafast and low memory footprint clustering (and ranking) of very large sets of atomistic or coarse-grained models of fixed length using residue profiles, or arbitrary macromolecular structures when using fragment profiles. At the same time, uQlust yields results on par with methods implying much higher computational cost in both model quality assessment and explicit clustering. A number of widely used methods and utilities for macromolecular structure analysis, including DSSP for protein secondary structure and solvent accessibility assignment [10], RNAview for RNA secondary structure and base-pair type assignment [11], and FragBag for fragment-based profile assignment [5], are implemented in uQlust and integrated into workflows for ranking and clustering without the need to use external programs. The code is freely available to the community and can be used in both batch and interactive modes, providing a versatile, efficient and easy-to-use toolkit for macromolecular structure exploration and analysis.

## Availability and requirements

The uQlust package is freely available under the GNU General Public License at https://github.com/uQlust. uQlust has been written in C# and should be easily portable between different operating systems. System independent pre-compiled executables that require .NET ver. 4.5 or higher, or Mono ver. 3.8 on 64-bit Windows or Linux operating systems, respectively, are provided with the distribution.

## Additional file

Additional file 1: Figure S1. 1D-jury algorithm for geometric consensus-based model ranking with contact map profiles. Three models (rows) of a hypothetical protein consisting of just 4 amino acid residues are considered, with the upper triangle of the inter-residue contact map (i,j) arranged as a linear profile. Black squares indicate contacts, while yellow squares indicate pairs of residues that are not in contact. The calculation of the score for the best scoring M2 model that corresponds to the consensus state at 5 (out of 6) profile positions proceeds red arrows. Note that a vector of state counts in each column of the profile can be precomputed in linear time, allowing one to account for all pairwise similarities without the need for a loop over pairs of models. **Figure S2.** Assessment of protein model selection on TASSER benchmark using uQlust: Hash (K,F) with different choices of the number of clusters K, and fraction of data included F. Low (averaged over all TASSER targets) RMSD of the top ranking model with respect to the best model available indicates better results. **Figure S3.** Comparison between full (RMSD-based average linkage) and uQlust:Tree (approximate) hierarchical clustering of coarse-grained structures obtained using CABS-flex server (Jamroz et al., 2013). Three initial conformations of troponin C are used to generate 3 distinct clusters (each containing 3,000 models, and marked by red, green and blue bars, respectively). **Figure S4.** Hierarchical clustering of ribosomal RNAs (blue – 16S, red – 23S, green - 5S) using the fragment-based RNA-FragBag profile, uQlust:Tree in conjunction with profile hashing (using the default number of microclusters) and cosine distance. **Table S1.** Structural profiles implemented in uQlust. For each profile, its type (as defined by the macromolecule it applies to, i.e., either protein or RNA), the source of state assignment, the number of states and the size (length) of the profile are reported. (PDF 260 kb)

## Abbreviations

CA: Carbon alpha (in a residue)
CASP: Critical assessment of structure prediction
CATH: Class, architecture, topology, homologous superfamily classification
CM: Contact map
LW: Leontis and Westhof (base pair classification)
MQA: Model quality assessment
NC: Number of contacts
PDB: Protein Data Bank
RMSD: Root mean square deviation
SA: Solvent accessibility
SS: Secondary structure
