# Supplementary material for: UQlust: combining profile hashing with linear-time ranking for efficient clustering and analysis of big macromolecular data
Source: BMC Bioinformatics. 2016 Dec 28;17:546. doi: 10.1186/s12859-016-1381-2 (PMC5198500; doi:10.1186/s12859-016-1381-2)
Supplement: Additional file 1: Figure S1. — 1D-jury algorithm for geometric consensus-based model ranking with contact map profiles. Three models (rows) of a hypothetical protein consisting of just 4 amino acid residues are considered, with the upper triangle of the inter-residue contact map (i,j) arranged as a linear profile. Black squares indicate contacts, while yellow squares indicate pairs of residues that are not in contact. The calculation of the score for the best scoring M2 model that corresponds to the consensus state at 5 (out of 6) profile positions proceeds red arrows. Note that a vector of state counts in each column of the profile can be precomputed in linear time, allowing one to account for all pairwise similarities without the need for a loop over pairs of models. Figure S2. Assessment of protein model selection on TASSER benchmark using uQlust: Hash (K,F) with different choices of the number of clusters K, and fraction of data included F. Low (averaged over all TASSER targets) RMSD of the top ranking model with respect to the best model available indicates better results. Figure S3. Comparison between full (RMSD-based average linkage) and uQlust:Tree (approximate) hierarchical clustering of coarse-grained structures obtained using CABS-flex server (Jamroz et al., 2013). Three initial conformations of troponin C are used to generate 3 distinct clusters (each containing 3,000 models, and marked by red, green and blue bars, respectively). Figure S4. Hierarchical clustering of ribosomal RNAs (blue – 16S, red – 23S, green - 5S) using the fragment-based RNA-FragBag profile, uQlust:Tree in conjunction with profile hashing (using the default number of microclusters) and cosine distance. Table S1. Structural profiles implemented in uQlust. For each profile, its type (as defined by the macromolecule it applies to, i.e., either protein or RNA), the source of state assignment, the number of states and the size (length) of the profile are reported. (PDF 260 kb) [file 12859_2016_1381_MOESM1_ESM.pdf]

Supplementary Materials

uQlust: Combining Profile Hashing with Linear-time Ranking for Efficient Clustering and Analysis of Big Macromolecular Data

Rafal Adamczak and Jarek Meller, BMC Bioinformatics

SM1: Efficient ranking and clustering of big macromolecular data

uQlust is a versatile package for efficient protein and RNA structure ranking and clustering, designed to overcome computational bottlenecks of current methods in the context of large scale molecular simulations. uQlust can be used for Markov state type conformational space partitioning by clustering, or model quality assessment for de novo prediction methods by ultrafast geometric consensus (clustering)-based ranking.

In particular, uQlust enables a low memory footprint (approximate) hierarchical clustering that achieves effective linear scaling for large data sets. Time and memory complexity is drastically reduced compared to traditional approaches by projecting 3D structures into a suitable 1D structural profile (an approximation often considered in this context), and importantly, by combining profile hashing with a linear time geometric consensus ranking algorithm, as discussed in the main manuscript.

Here, some of the key concepts are briefly reviewed, while examples of typical workflows on both protein and RNA data sets are used to further illustrate the performance and versatility of uQlust.

SM2: 1D-Jury geometric consensus ranking with implicit structure-to-structure comparison

Geometric consensus-based model ranking with 1D-jury (Adamczak and Meller, 2011) has been devised to provide a linear time solution accounting for similarity between all pairs of alternative 3D structures of the same macromolecule projected into a suitable 1D structural profile. This concept is illustrated here by introducing a linear time complexity approximation to the PconsD, as an example of a successful model quality assessment method that speeds up model-to-model comparison by considering their 2D distance matrix representation (Skwark and Elofsson, 2013).

A distance matrix element, d_{ij}, i.e., the distance between residues *i* and *j*, respectively, can be projected into binary contact map by using an appropriate threshold, e.g., $d_{ij} <$ 8.5 Ang. Note that more states could be introduced without the loss of generality by considering intermediate distances. However, as shown in the main manuscript, a binary projection works quite well. The upper triangle of such defined contact map can be rolled into a binary vector that constitutes a 1D structural profile. Figure 1S illustrates that for the hypothetical case of a protein with four amino acid residues (diagonal elements are omitted).

The state count (here for two states) vector is first precomputed in linear time with respect to the number of models, providing the count of black (contact) and yellow (no contact) states across all models in the set for each column (*i*,*j*). Subsequently, for each model, one can compute the 1D-jury geometric consensus score by summing up the number of models in the same state in each column, assigning higher scores to models that share contacts (in general substructures) with other models in the set. Thus, while assessing all pairwise similarities, 1D-jury does not require a loop over

the pairs of models and hence it provides a linear time complexity approximation to quadratic methods, such as PconsD (see Table 1 in the main article).

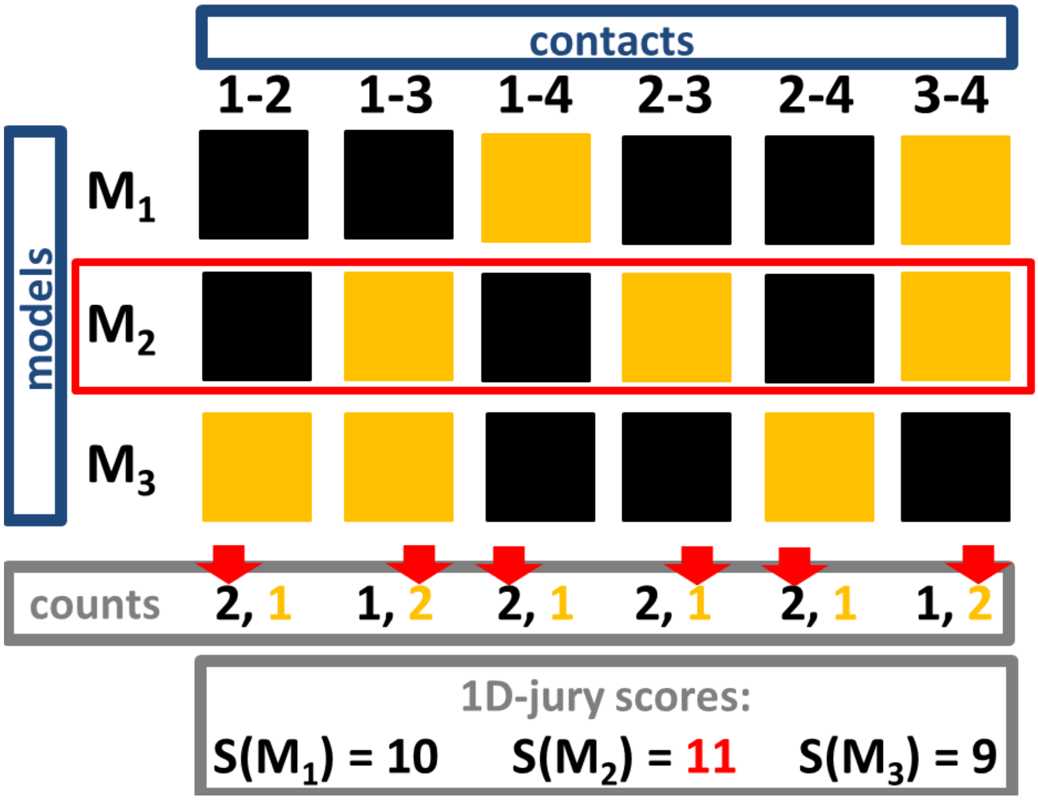

Figure 1S: 1D-jury algorithm for geometric consensus-based model ranking with contact map profiles. Three models (rows) of a hypothetical protein consisting of just 4 amino acid residues are considered, with the upper triangle of the inter-residue contact map (i,j) arranged as a linear profile. Black squares indicate contacts, while yellow squares indicate pairs of residues that are not in contact. The calculation of the score for the best scoring M2 model that corresponds to the consensus state at 5 (out of 6) profile positions proceeds red arrows. Note that a vector of state counts in each column of the profile can be precomputed in linear time, allowing one to account for all pairwise similarities without the need for a loop over pairs of models.

Note that in addition to providing a strictly linear consensus ranking algorithm, 1D-jury can be used to efficiently identify natural centroids for cluster of models sharing common substructures, with applications to enhanced K-means and micro-cluster aggregation. In particular, by combining 1D-jury with profile hashing, uQlust enables an approximate hierarchical clustering approach that achieves effective linear scaling for large data sets of macromolecular structures represented as 1D profiles (although it is not strictly linear in this case). Note also that 1D-jury can generalized to arbitrary structures (of any length) as long as they can be efficiently projected into fixed length structural profiles, such as fragment frequency profiles used by FragBag (Budowski-Tal et al., 2010).

SM3: Clustering heuristics in uQlust

Approximate hierarchical clustering in uQlust starts from an initial data slicing into micro-clusters (sets of structures with identical hashing keys) and proceeds to hierarchically aggregate data. In addition, several methods for ultrafast direct data partitioning into K clusters (including F fraction of all data points) are implemented in uQlust as a profile hashing-based alternative to K-means approaches. These efficient clustering heuristics that utilize profile hashing are referred to as:

- uQlust:Hash(K,F) that aggregates data into K clusters (comprising F% of data) by simply tuning-up the granularity of hashing keys;
- uQlust:Rpart(K,F) that uses a 1D-Jury reference-based partitioning of data while changing the radius of clustering to achieve K target cluster with F% of data;
- uQlust:Tree hierarchical clustering that proceeds to aggregate the initial hashing (or reference-based) micro-clusters using Hamming, cosine (or RMSD when applicable) distance.

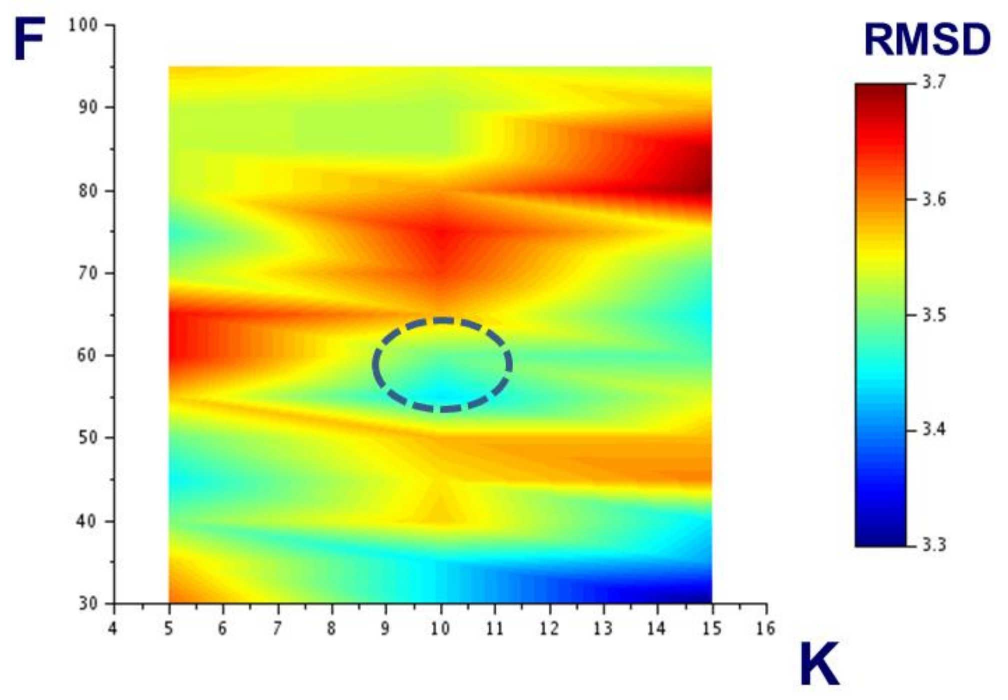

Figure 2S: Assessment of protein model selection on TASSER benchmark using uQlust:Hash(K,F) with different choices of the number of clusters K, and fraction of data included F. Low (averaged over all TASSER targets) RMSD of the top ranking model with respect to the best model available indicates better results.

The adjustable parameters K and F need to be provided by the user, with defaults set to K=10 and F=60% in most cases. These default values have been tested over a range of applications, including CASP and TASSER benchmarks for modeling quality assessment and the selection of best models by using centroids of top K clusters. As can be seen in Figure 2S, the ability to exclude a fraction of data (100%-F) that can represent many small clusters, provides an additional flexibility and contributes to the

fact that uQlust heuristics in general outperform significantly K-means approaches pm MQA benchmarks (see the main text).

It should be also noted that uQlust includes enhanced implementations of traditional hierarchical clustering and K-means methods (with 1D-jury based centroids – see section SM2) that can be applied in conjunction with commonly used 3D structure-to-structure comparison approaches and either global RMSD or local (substructure) MaxSub distance/similarity measures. This enables direct comparison of traditional techniques with profile hashing-based heuristics (on problem of smaller size when the computational cost of the former is not prohibitive).

SM4: Structural profiles available in uQlust

Each of the above heuristics can be applied in conjunction with any of the 1D structural profiles listed in Table 1S. These build in profiles enable clustering and analysis of both proteins and RNAs, while providing granularity and structural resolution that can be tuned up for specific applications. In addition to residue level profiles applicable to models of the same macromolecule, uQlust can also be used in conjunction with fragment-based (or other suitable profiles, including an arbitrary user defined profile) for ultrafast comparison, similarity search and clustering of arbitrary structures, e.g., enabling clustering and analysis of the entire PDB.

Table 1S. Structural profiles implemented in uQlust. For each profile, its type (as defined by the macromolecule it applies to, i.e., either protein or RNA), the source of state assignment, the number of states and the size (length) of the profile are reported.

Name	Type	Source	Number of States	Size
SS-SA	Prot	uQlust:DSSP	N_{SS} * N_{SA}	N_{res}
CA(SS)-NC(SA)	Prot	uQlust	N_{PSS} * N_{Cont}	N_{res}
CA-CM	Prot	uQlust	2	$N_{res}(N_{res}+1)/2$
FragBag	Prot	uQlust:FragBag	Max frequency	N_{frag}
RNA-SS-LW	RNA	uQlust:RNAview	N_{SS} * N_{LW}	N_{base}
RNA-SS-TA	RNA	DSSR	N_{SS} * N_{TA}	N_{base}
RNA-P-CM	RNA	uQlust	2	$N_{base}(N_{base}+1)/2$
RNA-FragBag	RNA	uQlust	Max frequency	N_{frag}
User generated	Prot/RNA	User defined	User defined	User defined

Depending on the application, profiles may be tuned and refined by the user by changing the level of coarse-graining (of residue states), combining different profiles, setting weights to emphasize the importance of certain states (e.g., secondary structure vs. solvent accessibility), or by changing the fragment library to be used in the case of FragBag type fragment frequency type profiles. Note, that unlike other profiles listed here, RNA-SS-TA is not computed internally and requires the DSSR utility to provide secondary structure and torsional angle state assignment for RNAs.

SM4: Predefined workflows and examples of results

Predefined workflows allow one to start with a default setup, where an appropriate profile and clustering parameters are selected for the problem at hand. The results of several such problems, including the clustering of coarse-grained models of three distinct conformers of troponin C (PDB structures 1TNX, 1TNW and 1YV0, length

162 aa) using the CA-CM profile, fragment-based clustering of about 100,000 protein chains from PDB using the FragBag profile, and fragment-based clustering (using the RNA-FragBag profile) of 5S, 16S and 23S ribosomal RNAs are discussed in the main text and are illustrated here.

A. Traditional hierarchical clustering using average linkage (PGMA) and RMSD.

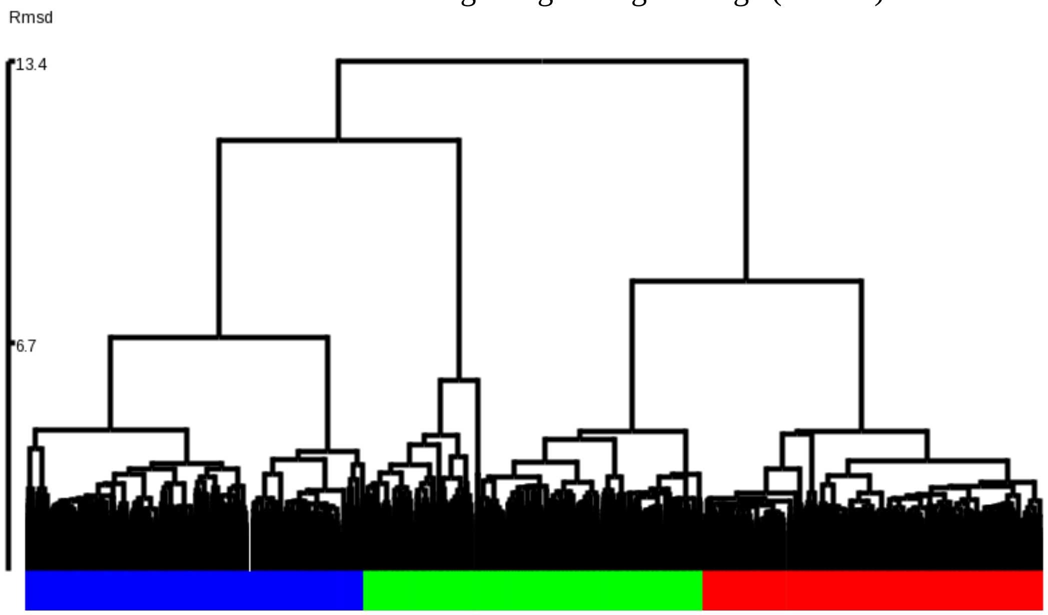

B. Profile hashing-based uQlust:Tree with the Hamming distance.

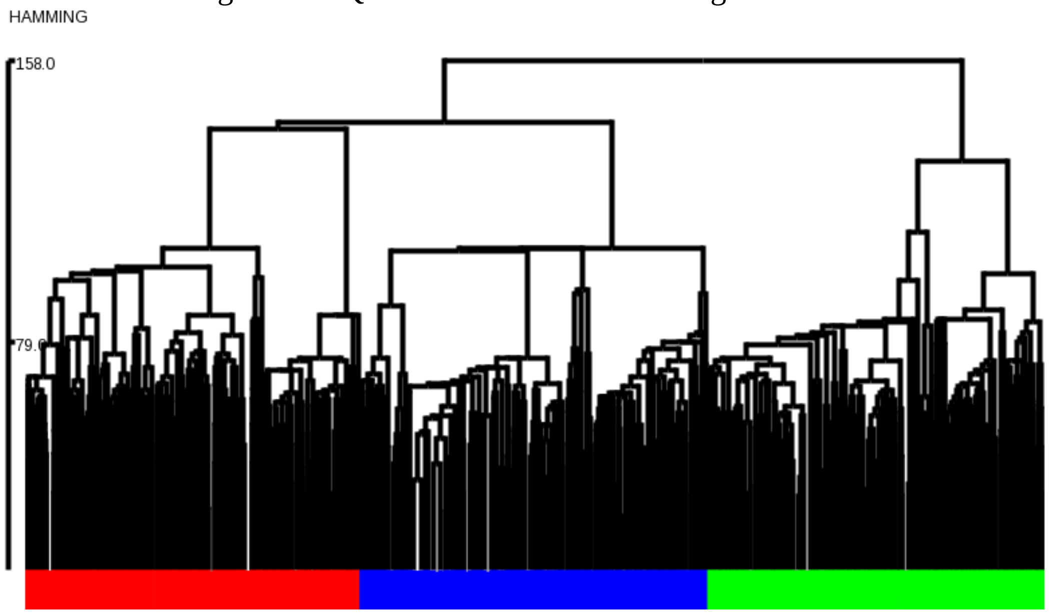

Figure 3S: Comparison between full (RMSD-based average linkage) and uQlust:Tree (approximate) hierarchical clustering of coarse-grained structures obtained using CABS-flex server (Jamroz et al., 2013). Three initial conformations of troponin C are used to generate 3 distinct clusters (each containing 3,000 models, and marked by red, green and blue bars, respectively).

Figure 3S compares the results for 9,000 structures in the troponin C benchmark (of Table 3 in the main text) using full, RMSD-based hierarchical clustering (panel A) vs.

approximate uQlust:Tree approach, using the CA-CM profile and Rpart(1000,100) to define initial micro-clusters, in conjunction with 1D-jury centroids and Hamming distance-based micro-cluster aggregation. It should be noted that while top 3 clusters are aggregated differently (with uQlust:Tree providing the correct solution in this case), the overall consistency between these two approaches is very high, as indicated by the Rand index of 0.99 at the level of 5 clusters.

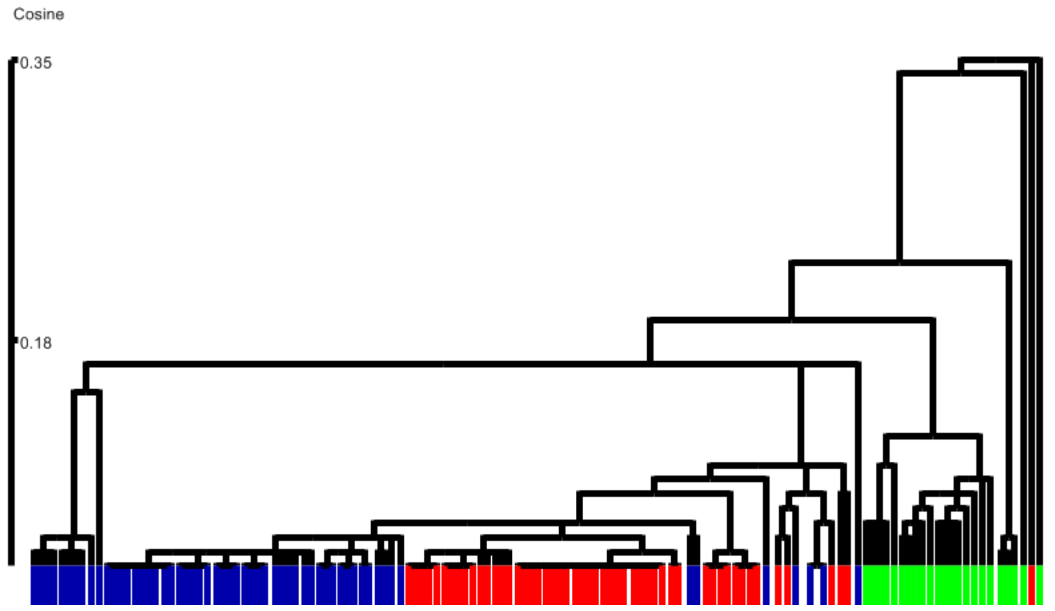

Figure 4S: Hierarchical clustering of ribosomal RNAs (blue – 16S, red – 23S, green - 5S) using the fragment-based RNA-FragBag profile, uQlust:Tree in conjunction with profile hashing (using the default number of microclusters) and cosine distance.

Figure 4S, on the other hand, illustrates hierarchical clustering of ribosomal RNAs using a fragment-based profile library of 92 coarse-grained 5-mer backbone (phosphorus atom) RNA fragments, derived from the RNA05 set of representative RNA structures (Humphris-Narayanan and Pyle, 2012). Such derived fragment were subsequently clustered by using uQlust:K-means and RMSD. The resulting custom library is used as a basis to define the RNA-FragBag fragment-based profile that is then applied in conjunction with uQlust:Tree and cosine distance to ribosomal RNAs, demonstrating high concordance with the three classes included.

Finally, Figure 2 in the main text illustrates the use of uQlust in conjunction with the FragBag structural motif frequency profile (Budowski-Tal et al., 2010) for analysis and clustering of arbitrary structures (of different length). This approach is put to the test by performing hierarchical clustering of about 98,000 protein chains from the PDB that were assigned no more than 1 CATH structural annotation. uQlust:Tree with the FragBag profile, Rpart(10000,90) micro-clusters and cosine similarity measure is used. Because CATH contains over thousand folds, a large number (10,000) of micro-clusters needs to be induced for these highly granular data. As can be seen from the Figure, the three main classes of proteins (alpha, alpha-beta and beta) cluster largely together, although a fraction of beta structures clusters with the alpha class, revealing limitations of a simple fragment frequency profile.

For references and acronyms, please see the main text. The uQlust package is available for downloading (including the source code and examples of standard workflows) at https://github.com/uQlust/uQlust-ver2.0. For further details, please see the User Manual also included in the uQlust distribution.
